# Development and validation of a SHAP-interpretable GBM model for predicting postoperative recurrence of anal Fistula

**DOI:** 10.3389/fsurg.2026.1911496

**Published:** 2026-08-26

**Authors:** Yunhao Zhou, Dawei Wang, Min Tang, Shaohua Huangfu, Xueping Zheng

**Affiliations:** 1 Colorectal Disease Center, Nanjing Hospital of Chinese Medicine Affiliated to Nanjing University of Chinese Medicine, Nanjing, Jiangsu Province, China; 2 Guang’anmen Hospital, China Academy of Chinese Medical Sciences, Beijing, China; 3 Shanghai University of Traditional Chinese Medicine, Shanghai, China

**Keywords:** anal fistula, gradient boosting machine, machine learning, postoperative recurrence, shap

## Abstract

**Background:**

This study aimed to develop and interpret a machine learning model for predicting postoperative recurrence of anal fistula using routine laboratory indicators and inflammation-related indices.

**Methods:**

A total of 2,214 patients who underwent fistulectomy were included. Patients from wards 5, 11, 12, 13 and 14 (*n* = 1,772) were divided by stratified random sampling according to recurrence status into training (*n* = 1,242) and testing (*n* = 530) cohorts. Patients from wards 15 and 16 (*n* = 442), which were managed by separate clinical teams, were reserved as a ward-based internal validation cohort. Univariate and multiple fistula tracts. analyses were performed to identify recurrence-associated factors, and LASSO regression was used for feature selection. Multiple machine learning models were developed and compared, including logistic regression, support vector machine, GBM, neural network, XGBoost, AdaBoost, LightGBM, and CatBoost. Model performance was assessed using ROC curves, calibration curves, decision curve analysis, and classification metrics. SHAP analysis was applied for model interpretation.

**Results:**

Multivariate logistic regression analysis showed that WBC, RBC, hs-CRP, and NCR were independent predictors of recurrence. LASSO regression selected 11 variables for model development. Among the candidate models, GBM demonstrated the most balanced predictive performance and was therefore selected as the final model. The AUCs of GBM in the training, testing, and validation sets were 0.777, 0.784, and 0.712, respectively. Calibration curves showed acceptable agreement between predicted and observed risks, while decision curve analysis indicated potential clinical benefit within low-to-moderate threshold probability ranges. SHAP analysis identified age, WBC, RBC, NCR, and hs-CRP as the main contributors to model prediction. Restricted cubic spline analysis revealed a significant nonlinear association between NCR and recurrence risk.

**Conclusion:**

WBC, RBC, hs-CRP, and NCR were independently associated with postoperative recurrence of anal fistula. The LASSO-based GBM model demonstrated stable predictive performance and acceptable clinical utility. Routine hematological parameters and inflammation-related indices, particularly NCR, may support individualized recurrence risk stratification and postoperative follow-up.

## 1 Introduction

Anal fistula is a common anorectal disorder characterized by an abnormal tract connecting the anal canal or rectum to the perianal skin (1). Although surgery remains the standard treatment, postoperative recurrence is still considered a major clinical challenge. Postoperative recurrence may result in repeated procedures, delayed wound healing, increased healthcare costs, and reduced quality of life (2). The primary goal of treatment is to ensure adequate drainage while preserving sphincter integrity, thereby reducing the risk of recurrence (3). Recurrence rates after surgical repair of anal fistula have been reported to range from 5.9% to 25.4%, underscoring the clinical burden of postoperative recurrence (4). Nevertheless, accurate individualized prediction of postoperative recurrence remains challenging in routine clinical practice.

Most existing studies have focused on anatomical and operative factors (5), whereas the contribution of systemic inflammatory status remains incompletely understood. Routine blood cell parameters and inflammatory markers are readily available preoperatively and may provide clinically useful information for recurrence risk assessment. In recent years, composite inflammation-related indices, including systemic immune-inflammation index (SII), systemic inflammation response index (SIRI), aggregate index of systemic inflammation (AISI), lymphocyte-to-C-reactive protein ratio (LCR), and neutrophil-to-C-reactive protein ratio (NCR), have been used to reflect systemic inflammatory and immune status in various clinical settings (5–9). Nevertheless, their value in predicting postoperative recurrence of anal fistula has not been adequately investigated.

Traditional regression analyses are useful for identifying risk factors; however, their ability to model nonlinear effects and interactions remains limited. Machine learning approaches may improve predictive performance by integrating multiple clinical and laboratory variables and capturing complex nonlinear relationships. Meanwhile, model interpretability remains essential for clinical application. Shapley additive explanations (SHAP) analysis can be used to quantify the contribution of each variable to model output, whereas restricted cubic spline (RCS) analysis can be applied to characterize nonlinear associations between continuous predictors and outcomes.

The present study was designed to identify factors associated with postoperative recurrence of anal fistula and to develop an interpretable machine learning prediction model based on routinely available clinical and laboratory variables. Logistic regression and least absolute shrinkage and selection operator (LASSO) regression were applied for risk factor identification and feature selection, respectively. Multiple machine learning models were subsequently constructed and compared, and the final model was interpreted using SHAP analysis. In addition, RCS analysis was performed to evaluate the nonlinear association between NCR and the risk of recurrence.

## 2 Materials and methods

### 2.1 Data source

A total of 2,954 patients admitted with a diagnosis of anal fistula to Nanjing Hospital of Chinese Medicine, affiliated with Nanjing University of Chinese Medicine, between April 2023 and April 2024 were screened. After the predefined eligibility criteria were applied and patients with incomplete clinical or follow-up data were excluded, 2,214 patients who underwent fistulectomy were included in the final analysis. All fistulectomies were performed by experienced colorectal surgeons in accordance with standardized institutional protocols.

**Figure 1:**
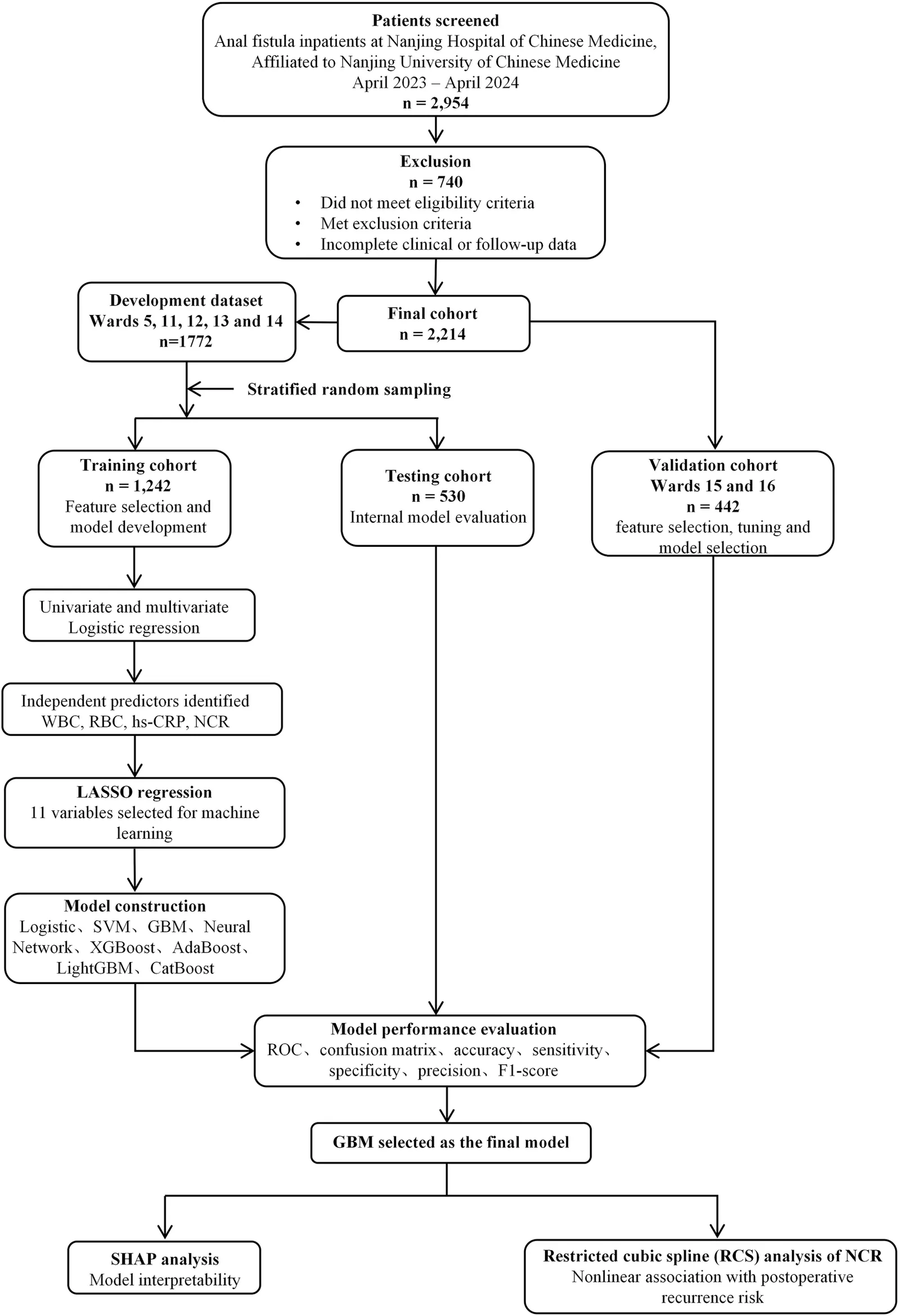
Study flowchart and technical roadmap. Study flowchart and model development and validation framework. A total of 2,954 patients admitted with anal fistula were screened, and 2,214 patients who underwent fistulectomy were included in the final analysis. Patients from wards 5, 11, 12, 13, and 14 constituted the development dataset (*n* = 1,772) and were divided by stratified random sampling according to postoperative recurrence status into a training cohort (*n* = 1,242) and a testing cohort (*n* = 530). Patients from wards 15 and 16 (*n* = 442), which were managed by separate clinical teams, were reserved as a ward-based internal validation cohort. Logistic regression and LASSO regression were used for variable selection, multiple machine learning models were developed and evaluated, and GBM was selected as the final model. SHAP analysis and restricted cubic spline analysis were further performed for model interpretation and assessment of the nonlinear association between NCR and postoperative recurrence risk.

The included patients were admitted to seven colorectal inpatient wards. Patients from wards 5, 11, 12, 13 and 14 constituted the development dataset, whereas patients from wards 15 and 16 constituted the ward-based validation cohort. Wards 15 and 16 were managed by clinical teams separate from those responsible for the development wards, although all wards belonged to the same institution and followed standardized institutional diagnostic, surgical, and postoperative management protocols (Figure 1).

Because this was a retrospective cohort study, all consecutive patients who met the predefined eligibility criteria during the study period were included, and the number of recruited patients was not determined by an *a priori* hypothesis-testing power calculation. The adequacy of the sample size for model development was assessed using the events-per-variable (EPV) approach (10). In our training dataset, the event rate is 0.15. Given that we plan to include 11 predictor variables, we set the EPV to 15 to increase statistical power, and we calculated the required sample size using the following formula:

$$\mathrm{Smaple}\;\mathrm{Size}=\frac{\mathrm{Number}\;\mathrm{of}\;\mathrm{Variables}\times \mathrm{EPV}}{\mathrm{Incidence}\;\mathrm{Rate}}=\frac{11\times 15}{0.15}=1100$$

Accordingly, the minimum required sample size was calculated as 15 × 11/0.15 = 1,100 patients, corresponding to at least 165 recurrence events. The training cohort included 1,242 patients, of whom 196 experienced postoperative recurrence, yielding an observed EPV of 17.8 for the 11 predictors retained in the final model. Therefore, the available training sample exceeded the minimum sample size estimated using the prespecified EPV criterion. The calculation was performed using R software, version 4.3.2.

Model development and validation were conducted in accordance with the TRIPOD (Transparent Reporting of a multivariable prediction model for Individual Prognosis Or Diagnosis) statement. Clinical data were extracted from the electronic medical record system. This study was approved by the Research Ethics Committee of Nanjing Hospital of Chinese Medicine, affiliated with Nanjing University of Chinese Medicine (approval No. 2026KY025-01), and conducted in accordance with the Declaration of Helsinki. Because anonymized retrospective data were analyzed, the requirement for informed consent was waived by the ethics committee.

### 2.2 Inclusion and exclusion criteria

#### 2.2.1 Inclusion criteria

Patients were eligible if they: (1) were aged ≥18 years; (2) were diagnosed with anal fistula according to established diagnostic criteria (11); and (3) underwent fistulectomy as the index surgical procedure.

### 2.2 Exclusion criteria

Patients were excluded if they had severe comorbidities, including malignancy, severe cardiopulmonary dysfunction, or hepatic or renal failure; an immunocompromised status, including long-term corticosteroid, immunosuppressive, or biologic therapy; inflammatory bowel disease, including Crohn's disease or ulcerative colitis; recent anorectal or colorectal surgery before the index admission; perianal skin infection, sebaceous gland infection, or other non-fistula-related perianal infections; coagulation disorders; severe active infections in other organs or systems; pregnancy or lactation; or incomplete clinical, laboratory, or follow-up data.

### 2.3 Study variables and outcome definitions

Clinical data were extracted from the electronic medical records of patients admitted with a diagnosis of anal fistula. The variables included demographic characteristics, lifestyle factors, comorbidities, fistula-related characteristics, and laboratory indicators. Demographic variables included age, sex, height, weight, and body mass index (BMI). Lifestyle factors included smoking status and alcohol consumption. Comorbidities included a history of hypertension and diabetes mellitus. Fistula-related characteristics included the presence of high anal fistula (12) and multiple fistula tracts.

Laboratory indicators were obtained from routine blood tests and inflammatory markers measured within 24 h before surgery. These included white blood cell count (WBC), red blood cell count (RBC), platelet count (PLT), hemoglobin (Hb), neutrophil percentage and absolute neutrophil count (Neut), lymphocyte count, monocyte count, eosinophil count, basophil count, mean corpuscular volume (MCV), mean platelet volume (MPV), and high-sensitivity C-reactive protein (hs-CRP). Blood samples were collected from peripheral venous blood, and all laboratory analyses were performed by the hospital laboratory department according to standardized protocols. Inflammation-related composite indices were further calculated, including the neutrophil-to-lymphocyte ratio (NLR), platelet-to-lymphocyte ratio (PLR), SII, SIRI, AISI (13), LCR, NCR (14).

The primary outcome was recurrence of anal fistula after the index fistulectomy. Recurrence was defined as the reappearance of anal fistula-related symptoms or signs during follow-up, including a persistent or recurrent external opening, purulent discharge, abscess formation, or clinical or imaging evidence of fistula recurrence after fistulectomy. Patients were followed for 2 years after fistulectomy and were classified into recurrence and non-recurrence groups according to their recurrence status.

### 2.4 Data preprocessing and variable selection

After patients with incomplete clinical or follow-up data were excluded, no missing values remained in the final dataset. Outliers in continuous variables, including NCR, were retained because they were considered potentially clinically meaningful. Continuous variables were standardized by z-score transformation to improve comparability across variables measured on different scales. Binary categorical variables were numerically encoded, with 0 denoting the reference category and 1 denoting the comparison category. Thirty-three candidate features were included in the final dataset, comprising 8 categorical variables and 25 continuous variables. For risk factor identification, univariate and multivariable logistic regression analyses were initially performed to evaluate associations between candidate variables and postoperative recurrence of anal fistula. WBC, RBC, hs-CRP, and NCR were identified as independent predictors of postoperative recurrence. Subsequently, LASSO regression was applied for further feature selection. The optimal penalty parameter *λ* was determined using cross-validation, and variables with nonzero coefficients were retained for model development. A total of 11 variables were selected for machine learning model development, including history of hypertension, age, neutrophil percentage (Neut_pre), Neut, eosinophil count, MPV, WBC, RBC, hs-CRP, AISI, and NCR.

### 2.5 Model development and performance evaluation

Before model development, the final cohort was partitioned according to inpatient ward. Patients admitted to wards 5, 11, 12, 13, and 14 constituted the development dataset (*n* = 1,772). This dataset was divided by stratified random sampling according to postoperative recurrence status into a training cohort (*n* = 1,242) and a testing cohort (*n* = 530), thereby maintaining comparable recurrence proportions between the two cohorts. Patients admitted to wards 15 and 16 (*n* = 442), which were managed by clinical teams separate from those responsible for the development wards, were reserved as a ward-based validation cohort. The training cohort was used for variable selection and model development, the testing cohort for model tuning and internal evaluation, and the validation cohort for assessing the generalizability of the final model. Using the 11 LASSO-selected variables, multiple prediction models were developed, including logistic regression, support vector machine (SVM), gradient boosting machine (GBM), neural network, extreme gradient boosting (XGBoost), adaptive boosting (AdaBoost), light gradient boosting machine (LightGBM), and category boosting (CatBoost). Model performance was evaluated using the area under the receiver operating characteristic curve (AUC), accuracy, sensitivity, specificity and F1-score. Confusion matrices were generated to evaluate the classification performance of each model. The final model was selected according to its overall predictive performance across the training, testing, and validation cohorts. To enhance model interpretability, was applied to the final model to quantify the contribution of each predictor to the model output. SHAP summary plots, feature importance plots, and individual-level explanation plots were generated to provide both global and local model explanations.

### 2.6 Statistical analysis

All data analyses and graphical visualizations for model construction were conducted using R software (version 4.3.2). Quantitative data with normal distribution were presented as mean ± standard deviation, and differences between groups were assessed using the independent-samples *t*-test. Non-normally distributed data were expressed as median (P25, P75) and compared using the Mann–Whitney *U*-test. Categorical variables were expressed as frequencies and percentages [n (%)] and compared using the chi-squared (*χ*^2^) test. Statistical significance was defined as a *p* < 0.05.

## 3 Results

### 3.1 Baseline characteristics in the training cohort

In the training cohort, 1,242 patients were included, comprising 1,046 patients in the non-recurrence group and 196 patients in the recurrence group. Compared with the non-recurrence group, patients in the recurrence group had significantly higher WBC, RBC, and hs-CRP levels, whereas LCR and NCR were significantly lower. The proportion of multiple fistula tracts was higher in the recurrence group than in the non-recurrence group, although the difference was marginally significant (Table 1).

**Table 1:** Baseline characteristics of patients in the training cohort.

Characteristic	Non-recurrence group (*N* = 1,046)	Recurrence group (*N* = 196)	*p*-value
	No	Yes	p
N	1,046	196	
Sex = 1 (%)	892 (85.3)	167 (85.2)	1
Historyofdiabetesmellitus = 1 (%)	63 (6.0)	14 (7.1)	0.663
Historyofhypertension = 1 (%)	211 (20.2)	29 (14.8)	0.099
Highanalfistula = 1 (%)	74 (7.1)	17 (8.7)	0.523
Multiplefistulatracts = 1 (%)	131 (12.5)	35 (17.9)	0.058
Alcohol = 1 (%)	98 (9.4)	20 (10.2)	0.816
Smoking = 1 (%)	61 (5.8)	16 (8.2)	0.28
Age [mean (SD)]	38.89 (12.11)	40.40 (12.26)	0.11
Height [mean (SD)]	172.43 (7.34)	172.95 (6.85)	0.356
Weight [mean (SD)]	77.18 (14.58)	77.60 (15.19)	0.715
BMI [mean (SD)]	25.87 (4.09)	25.84 (4.29)	0.947
Neut_pre [mean (SD)]	62.03 (9.67)	61.76 (10.13)	0.722
Neut [mean (SD)]	4.31 (1.94)	4.39 (2.18)	0.636
Mono_pre [mean (SD)]	6.03 (1.53)	6.22 (1.58)	0.115
Mono [mean (SD)]	0.41 (0.17)	0.43 (0.18)	0.166
Baso [mean (SD)]	0.03 (0.01)	0.03 (0.02)	0.94
Eos [mean (SD)]	0.14 (0.12)	0.16 (0.13)	0.156
MCV [mean (SD)]	89.62 (4.96)	89.08 (4.99)	0.16
MPV [mean (SD)]	10.28 (1.24)	10.39 (1.33)	0.255
Lymph [mean (SD)]	1.91 (0.63)	1.93 (0.70)	0.679
WBC [mean (SD)]	6.76 (3.43)	7.79 (3.46)	<0.001
RBC [mean (SD)]	4.83 (0.70)	4.97 (0.71)	0.008
PLT [mean (SD)]	231.97 (58.47)	234.41 (63.30)	0.596
Hb [mean (SD)]	146.07 (15.59)	144.61 (17.32)	0.235
hs-CRP [mean (SD)]	6.18 (10.37)	13.79 (21.79)	<0.001
NLR [mean (SD)]	2.59 (2.14)	2.65 (2.03)	0.728
PLR [mean (SD)]	135.24 (63.63)	137.16 (64.07)	0.699
SII [mean (SD)]	615.50 (595.58)	608.73 (461.26)	0.88
SIRI [mean (SD)]	1.18 (1.82)	1.23 (1.38)	0.717
AISI [mean (SD)]	288.01 (484.00)	289.12 (350.95)	0.976
LCR [mean (SD)]	0.63 (0.51)	0.49 (0.53)	<0.001
NCR [mean (SD)]	1.31 (1.01)	0.94 (0.86)	<0.001

### 3.2 Univariate and multivariate logistic regression analyses

Univariate logistic regression showed that multiple fistula tracts, WBC, RBC, hs-CRP, LCR, and NCR were significantly associated with postoperative recurrence of anal fistula. Multiple fistula tracts (OR = 1.52, 95% CI: 1.01–2.29, *p* = 0.045), higher WBC (OR = 1.07, 95% CI: 1.03–1.12, *p* < 0.001), RBC (OR = 1.29, 95% CI: 1.02–1.62, *p* = 0.03), and hs-CRP (OR = 1.03, 95% CI: 1.02–1.05, *p* < 0.001) were associated with increased recurrence risk, whereas higher LCR (OR = 0.53, 95% CI: 0.37–0.75, *p* < 0.001) and NCR (OR = 0.61, 95% CI: 0.49–0.74, *p* < 0.001) were associated with reduced risk.

Variables significant in univariate analysis were entered into the multivariate model. After adjustment, WBC (OR = 1.06, 95% CI: 1.02–1.11, *p* = 0.003), RBC (OR = 1.39, 95% CI: 1.07–1.81, *p* = 0.01), and hs-CRP (OR = 1.02, 95% CI: 1.01–1.04, *p* < 0.001) remained independent risk factors, whereas NCR remained independently associated with lower recurrence risk (OR = 0.56, 95% CI: 0.39–0.80, *p* = 0.001). Multiple fistula tracts and LCR were no longer significant after adjustment (Table 2).

**Table 2:** Univariate and multivariate logistic regression analyses of factors associated with postoperative recurrence of anal fistula.

name	desc	No (*N* = 1,046)	Yes (*N* = 196)	id	OR (univariable)	OR (multivariable)
Sex	0	154 (14.7%)	29 (14.8%)	Sex0		
	1	892 (85.3%)	167 (85.2%)	Sex1	0.99 (0.65–1.53, *p* = .979)	
Historyofdiabetesmellitus	0	983 (94%)	182 (92.9%)	Historyofdiabetesmellitus0		
	1	63 (6%)	14 (7.1%)	Historyofdiabetesmellitus1	1.20 (0.66–2.19, *p* = .551)	
Historyofhypertension	0	835 (79.8%)	167 (85.2%)	Historyofhypertension0		
	1	211 (20.2%)	29 (14.8%)	Historyofhypertension1	0.69 (0.45–1.05, *p* = .082)	
Highanalfistula	0	972 (92.9%)	179 (91.3%)	Highanalfistula0		
	1	74 (7.1%)	17 (8.7%)	Highanalfistula1	1.25 (0.72–2.16, *p* = .431)	
Multiplefistulatracts	0	915 (87.5%)	161 (82.1%)	Multiplefistulatracts0		
	1	131 (12.5%)	35 (17.9%)	Multiplefistulatracts1	1.52 (1.01–2.29, *p* = .045)	1.21 (0.78–1.88, *p* = .385)
Alcohol	0	948 (90.6%)	176 (89.8%)	Alcohol0		
	1	98 (9.4%)	20 (10.2%)	Alcohol1	1.10 (0.66–1.83, *p* = .715)	
Smoking	0	985 (94.2%)	180 (91.8%)	Smoking0		
	1	61 (5.8%)	16 (8.2%)	Smoking1	1.44 (0.81–2.55, *p* = .216)	
Age	Mean ± SD	38.9 ± 12.1	40.4 ± 12.3	Age	1.01 (1.00–1.02, *p* = .110)	
Height	Mean ± SD	172.4 ± 7.3	172.9 ± 6.9	Height	1.01 (0.99–1.03, *p* = .355)	
Weight	Mean ± SD	77.2 ± 14.6	77.6 ± 15.2	Weight	1.00 (0.99–1.01, *p* = .715)	
BMI	Mean ± SD	25.9 ± 4.1	25.8 ± 4.3	BMI	1.00 (0.96–1.04, *p* = .947)	
Neut_pre	Mean ± SD	62.0 ± 9.7	61.8 ± 10.1	Neut_pre	1.00 (0.98–1.01, *p* = .721)	
Neut	Mean ± SD	4.3 ± 1.9	4.4 ± 2.2	Neut	1.02 (0.94–1.10, *p* = .635)	
Mono_pre	Mean ± SD	6.0 ± 1.5	6.2 ± 1.6	Mono_pre	1.08 (0.98–1.19, *p* = .115)	
Mono	Mean ± SD	0.4 ± 0.2	0.4 ± 0.2	Mono	1.80 (0.78–4.13, *p* = .167)	
Baso	Mean ± SD	0.0 ± 0.0	0.0 ± 0.0	Baso	1.47 (0.00–31,600.23, *p* = .940)	
Eos	Mean ± SD	0.1 ± 0.1	0.2 ± 0.1	Eos	2.30 (0.73–7.31, *p* = .157)	
MCV	Mean ± SD	89.6 ± 5.0	89.1 ± 5.0	MCV	0.98 (0.95–1.01, *p* = .160)	
MPV	Mean ± SD	10.3 ± 1.2	10.4 ± 1.3	MPV	1.07 (0.95–1.21, *p* = .255)	
Lymph	Mean ± SD	1.9 ± 0.6	1.9 ± 0.7	Lymph	1.05 (0.83–1.33, *p* = .678)	
WBC	Mean ± SD	6.8 ± 3.4	7.8 ± 3.5	WBC	1.07 (1.03–1.12, *p* < .001)	1.06 (1.02–1.11, *p* = .003)
RBC	Mean ± SD	4.8 ± 0.7	5.0 ± 0.7	RBC	1.29 (1.02–1.62, *p* = .033)	1.39 (1.07–1.81, *p* = .013)
PLT	Mean ± SD	232.0 ± 58.5	234.4 ± 63.3	PLT	1.00 (1.00–1.00, *p* = .596)	
Hb	Mean ± SD	146.1 ± 15.6	144.6 ± 17.3	Hb	0.99 (0.99–1.00, *p* = .236)	
hs-CRP	Mean ± SD	6.2 ± 10.4	13.8 ± 21.8	hs-CRP	1.03 (1.02–1.05, *p* < .001)	1.02 (1.01–1.04, *p* < .001)
NLR	Mean ± SD	2.6 ± 2.1	2.7 ± 2.0	NLR	1.01 (0.95–1.08, *p* = .728)	
PLR	Mean ± SD	135.2 ± 63.6	137.2 ± 64.1	PLR	1.00 (1.00–1.00, *p* = .699)	
SII	Mean ± SD	615.5 ± 595.6	608.7 ± 461.3	SII	1.00 (1.00–1.00, *p* = .880)	
SIRI	Mean ± SD	1.2 ± 1.8	1.2 ± 1.4	SIRI	1.02 (0.94–1.10, *p* = .717)	
AISI	Mean ± SD	288.0 ± 484.0	289.1 ± 350.9	AISI	1.00 (1.00–1.00, *p* = .976)	
LCR	Mean ± SD	0.6 ± 0.5	0.5 ± 0.5	LCR	0.53 (0.37–0.75, *p* < .001)	1.72 (0.97–3.05, *p* = .061)
NCR	Mean ± SD	1.3 ± 1.0	0.9 ± 0.9	NCR	0.61 (0.49–0.74, *p* < .001)	0.56 (0.39–0.80, *p* = .001)

### 3.3 LASSO feature selection

**Figure 2:**
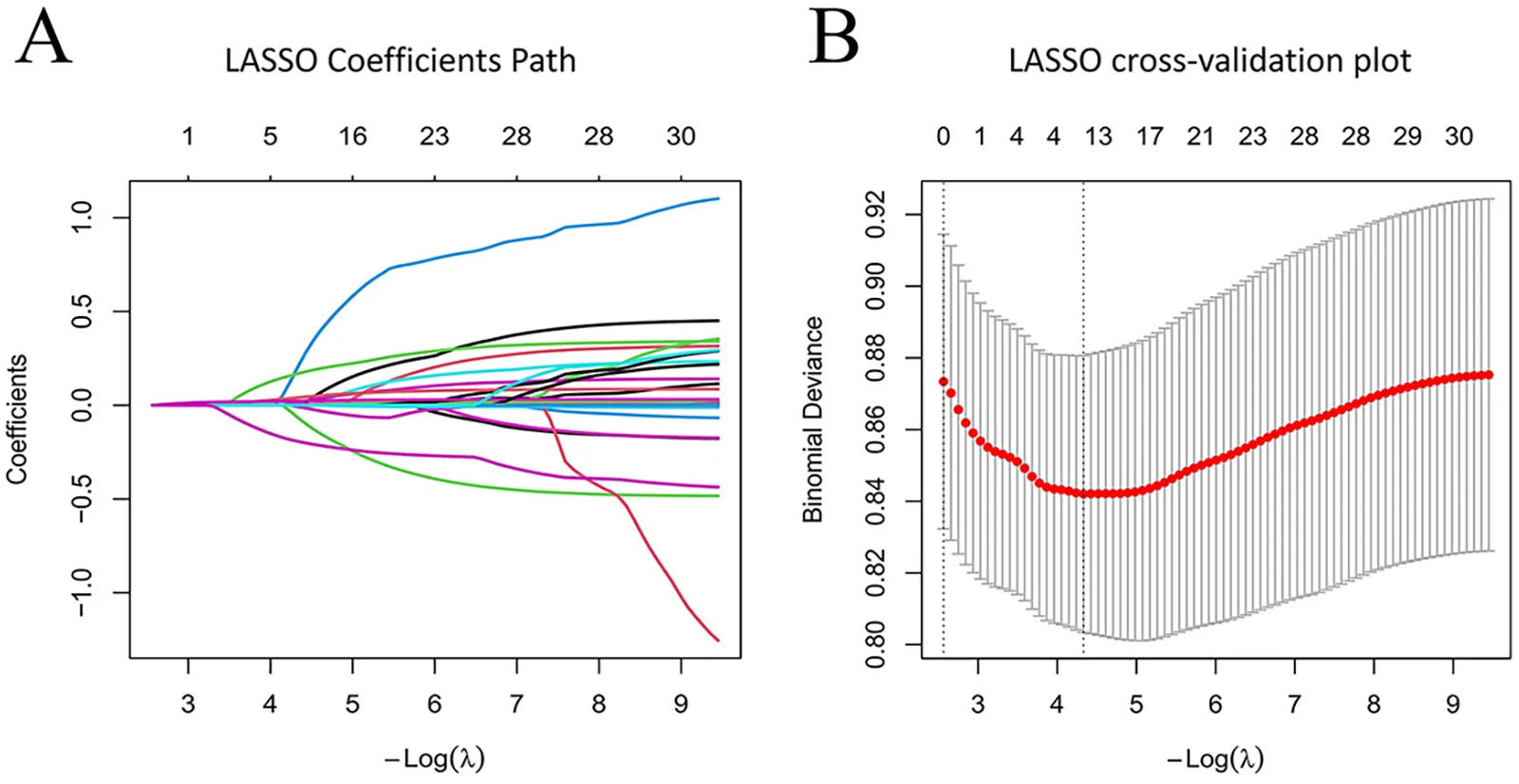
Variable selection using LASSO regression. **(A)** Coefficient profiles of the LASSO regression model. **(B)** Cross-validation plot for selection of the optimal penalty parameter (*λ*). LASSO, least absolute shrinkage and selection operator.

LASSO regression was applied to further select candidate variables for machine learning model development. As the penalty parameter increased, the coefficients of candidate variables progressively shrank toward zero (Figure 2A). The optimal *λ* value was determined using cross-validation (Figure 2B), and 11 variables with nonzero coefficients were retained: hypertension, age, Neut_pre, neutrophil count, eosinophil count, MPV, WBC, RBC, hs-CRP, AISI, and NCR. These variables were subsequently used for machine learning model development.

### 3.4 Machine learning model development and performance comparison

**Figure 3:**
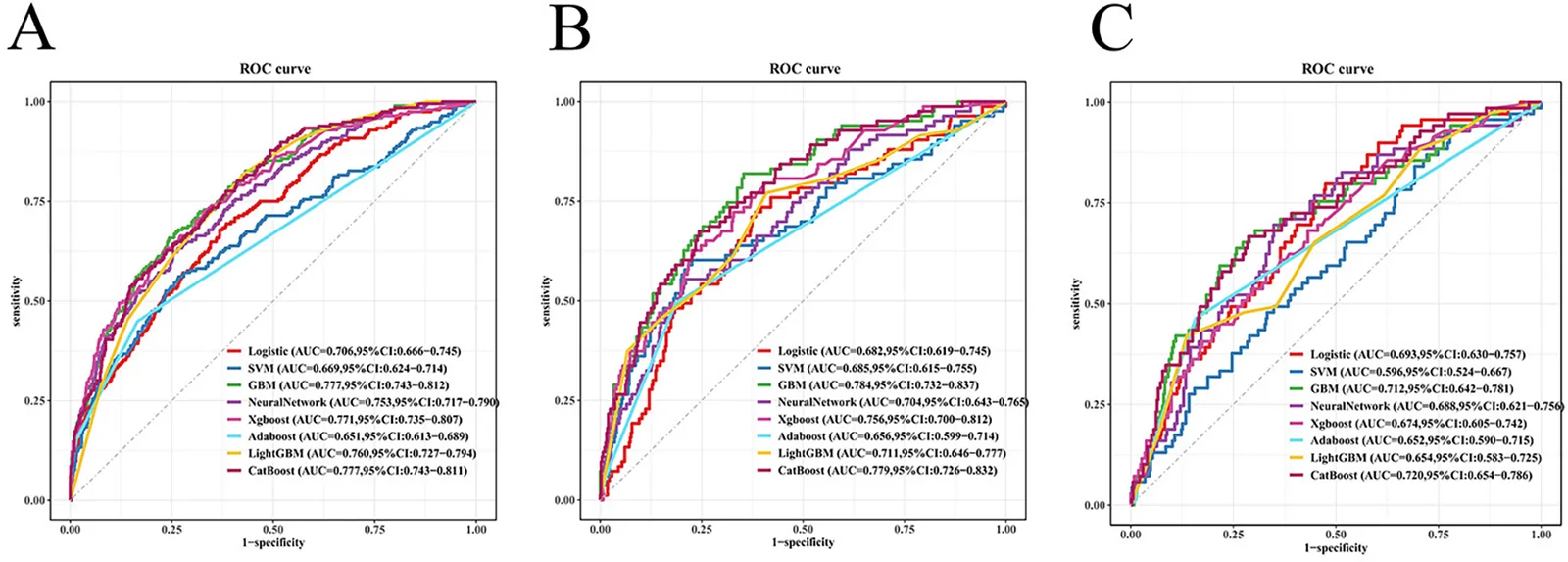
ROC curves of different prediction models. ROC curves of the prediction models in the training, testing, and validation cohorts. **(A)** Training cohort. **(B)** Testing cohort. **(C)** Validation cohort. ROC, receiver operating characteristic; AUC, area under the curve.

**Figure 4:**
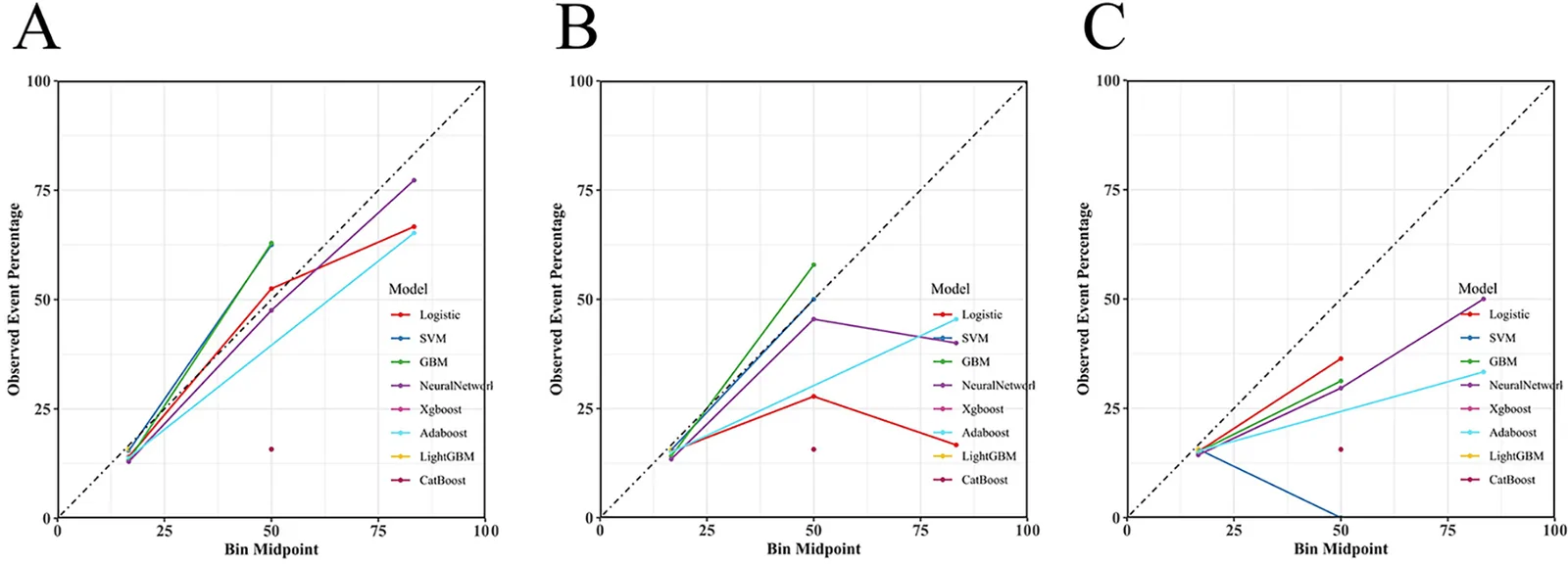
Calibration curves of different prediction models in the training, testing, and validation cohorts. **(A)** Training cohort. **(B)** Testing cohort. **(C)** Validation cohort. The dashed diagonal line represents perfect calibration.

**Figure 5:**
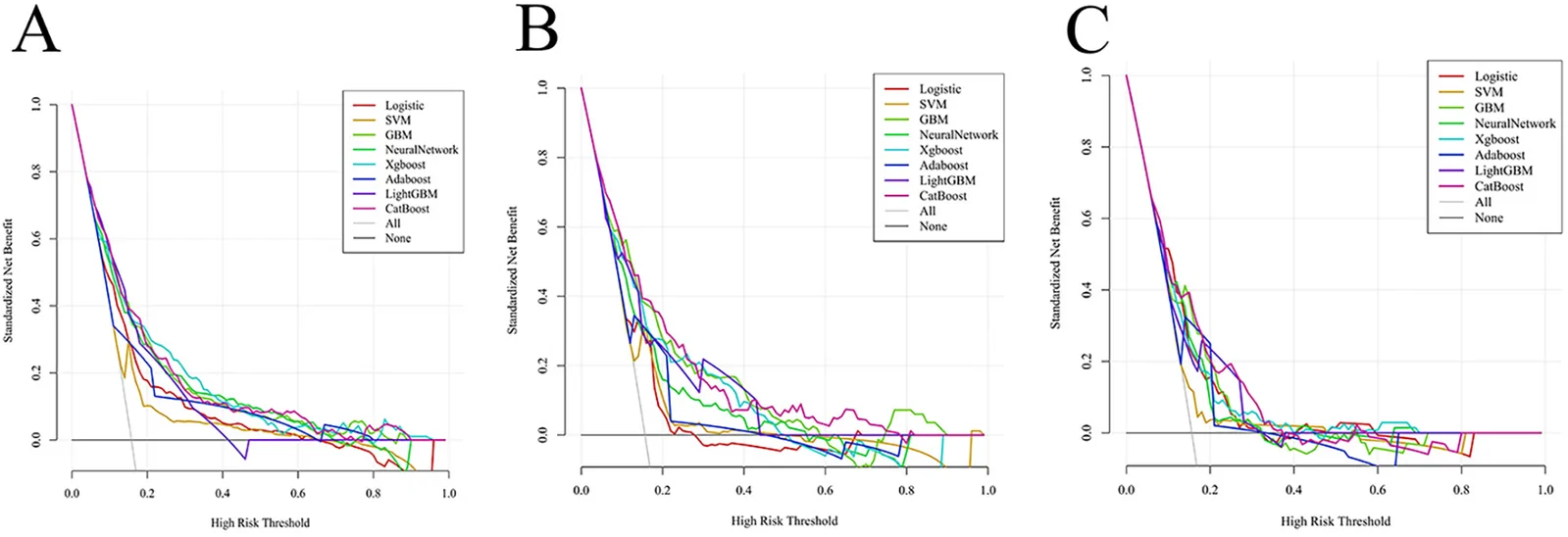
DCA of different prediction models in the training, testing, and validation cohorts. **(A)** Training cohort. **(B)** Testing cohort. **(C)** Validation cohort. The “All” and “None” lines represent the strategies of treating all patients as high risk and treating no patients as high risk, respectively.

Using the 11 LASSO-selected variables, eight prediction models were developed and evaluated in the training, testing, and validation cohorts using receiver operating characteristic (ROC) curves, calibration curves, decision curve analysis (DCA), and classification metrics. Among them, GBM showed favorable and stable performance, with AUCs of 0.777, 0.784, and 0.712 in the training, testing, and validation cohorts, respectively (Figure 3). Although CatBoost achieved a slightly higher validation AUC, GBM showed more balanced overall classification performance. In the three cohorts, the accuracy of GBM was 0.741, 0.745, and 0.724; sensitivity was 0.658, 0.663, and 0.638; specificity was 0.756, 0.761, and 0.740; and F1 values were 0.922, 0.924, and 0.917, respectively (Table 3). Calibration curves indicated acceptable agreement between predicted and observed recurrence risks (Figure 4), and DCA showed potential clinical net benefit across low-to-moderate threshold probabilities (Figure 5). Therefore, GBM was selected as the final prediction model.

**Table 3:** Performance comparison of different prediction models in the training, testing, and validation cohort.

Model	Training cohort					Testing cohort					Validation cohort				
	Threshold	Accuracy	Sensitivity	Specificity	F1	Threshold	Accuracy	Sensitivity	Specificity	F1	Threshold	Accuracy	Sensitivity	Specificity	F1
NA	NA	NA	NA	NA	NA	NA	NA	NA	NA	NA	NA	NA	NA	NA	NA
Logistic	0.149	0.639	0.679	0.632	0.913	0.149	0.630	0.723	0.613	0.923	0.149	0.633	0.638	0.633	0.904
SVM	0.155	0.706	0.561	0.733	0.899	0.155	0.679	0.602	0.694	0.904	0.155	0.667	0.406	0.716	0.867
GBM	0.154	0.741	0.658	0.756	0.922	0.154	0.745	0.663	0.761	0.924	0.154	0.724	0.638	0.740	0.917
NeuralNetwork	0.178	0.726	0.622	0.746	0.913	0.178	0.702	0.578	0.725	0.903	0.178	0.679	0.522	0.708	0.889
Xgboost	0.497	0.680	0.724	0.672	0.929	0.497	0.677	0.699	0.673	0.923	0.497	0.647	0.580	0.660	0.895
Adaboost	0.103	0.774	0.449	0.835	0.89	0.103	0.764	0.494	0.814	0.897	0.103	0.783	0.464	0.842	0.895
LightGBM	0.158	0.610	0.821	0.570	0.945	0.158	0.619	0.771	0.591	0.933	0.158	0.568	0.652	0.552	0.896
CatBoost	0.537	0.642	0.776	0.617	0.936	0.537	0.660	0.759	0.642	0.935	0.537	0.640	0.696	0.630	0.918

### 3.5 SHAP interpretation of the final GBM model

SHAP analysis was performed to interpret the final GBM model and quantify the contribution of each predictor. The SHAP importance plot showed that age had the highest mean absolute SHAP value, followed by WBC, RBC, NCR, hs-CRP, AISI, neutrophil count, Neut_pre, eosinophil count, hypertension, and MPV. Notably, WBC, RBC, hs-CRP, and NCR were also identified as independent predictors in multivariable logistic regression, supporting their stable contribution to recurrence prediction.

**Figure 6:**
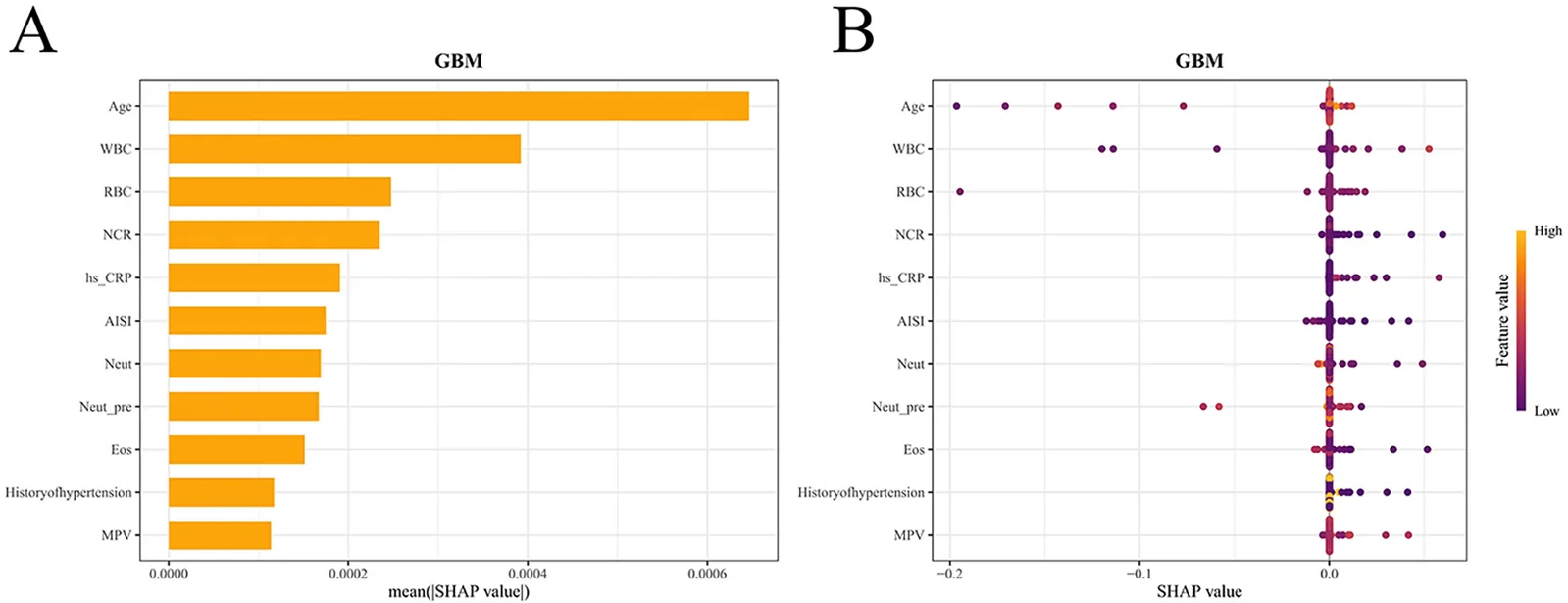
SHAP interpretation of the final GBM model. **(A)** SHAP importance plot showing the mean absolute SHAP value of each predictor. **(B)** SHAP beeswarm plot showing the distribution and direction of each predictor's effect on model output. SHAP, Shapley additive explanations; GBM, gradient boosting machine.

**Figure 7:**
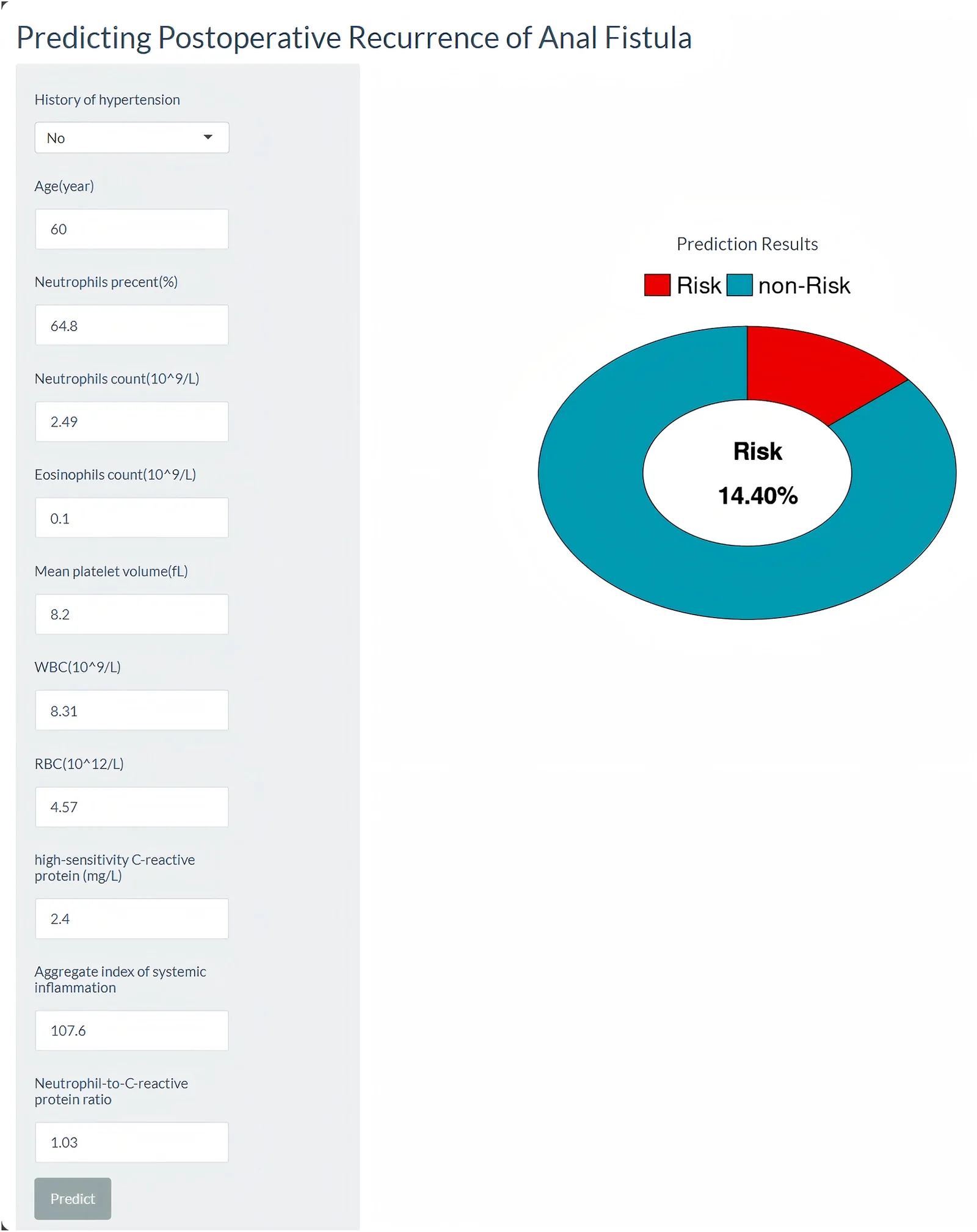
Web-based application for predicting postoperative recurrence of anal fistula. The final GBM model was deployed as an interactive Shiny application. Users can enter the 11 model predictors, and the application automatically calculates and graphically displays the individualized probability of postoperative recurrence. In the representative case shown, the predicted recurrence probability was 14.40%.

The SHAP beeswarm plot further illustrated the direction and distribution of each variable's effect on model output and suggested nonlinear effects for several predictors. Although age ranked first in SHAP importance, it was not an independent risk factor in multivariable analysis; therefore, its contribution should be interpreted as model-based predictive information rather than evidence of a causal association. Overall, SHAP analysis indicated that blood cell parameters and inflammation-related markers, particularly WBC, RBC, hs-CRP, and NCR, contributed substantially to predicting postoperative recurrence of anal fistula and improved the interpretability of the GBM model (Figure 6). To facilitate clinical implementation, we deployed the final GBM model as an interactive web-based application developed using the Shiny framework. The application allows users to enter the 11 predictors selected by LASSO regression, including history of hypertension, age, neutrophil percentage, neutrophil count, eosinophil count, mean platelet volume, WBC, RBC, hs-CRP, AISI, and NCR. After submission, the application automatically generates an individualized predicted probability of postoperative anal fistula recurrence and presents the result graphically. For the representative patient shown in Figure X, the predicted probability of postoperative recurrence was 14.40%. The application is intended to support individualized risk stratification and postoperative follow-up planning rather than replace clinical judgment (Figure 7).

### 3.6 Restricted cubic spline analysis of NCR

**Figure 8:**
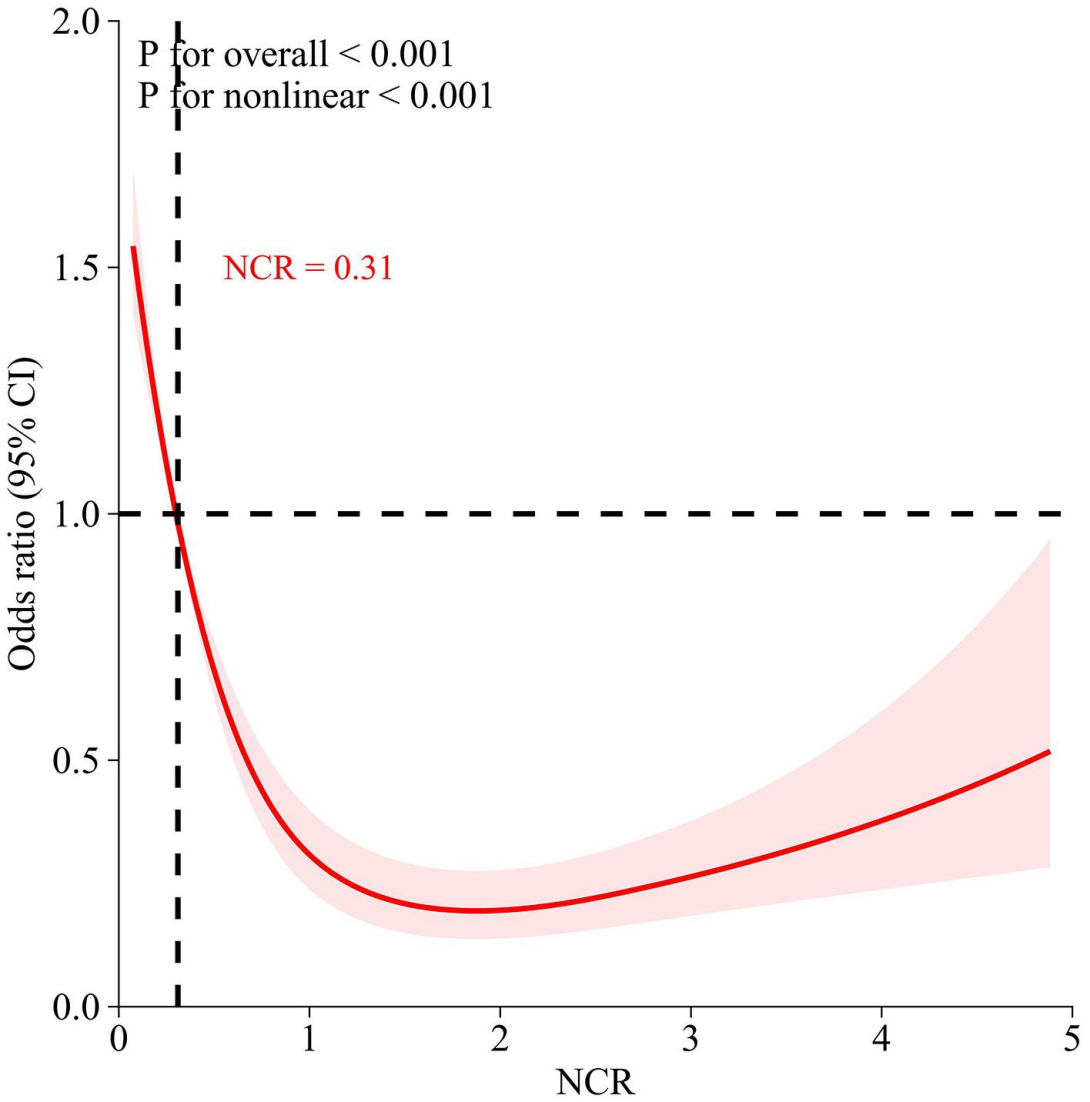
Restricted cubic spline analysis of the association between NCR and postoperative recurrence of anal fistula. The solid red line represents the estimated odds ratio, and the shaded area represents the 95% confidence interval. The horizontal dashed line indicates an odds ratio of 1. NCR, neutrophil-to-C-reactive protein ratio.

Restricted cubic spline analysis showed a significant nonlinear association between NCR and postoperative recurrence risk (*P* for overall <0.001; *P* for nonlinear <0.001). Recurrence risk decreased rapidly as NCR increased at lower levels, remained relatively low at intermediate levels, and increased slightly at higher levels, although the estimated odds ratio generally remained below 1 across higher NCR ranges (Figure 8). (Predicting Postoperative Recurrence of Anal Fistula.html).

Quartile analysis further supported this association. Compared with the lowest NCR quartile, higher quartiles were associated with significantly lower recurrence risk. This association remained stable after adjustment for age, sex, alcohol consumption, smoking status, and BMI. These findings suggest a nonlinear inverse association between NCR and recurrence risk and support the potential value of NCR for postoperative risk stratification in patients with anal fistula (Table 4). The baseline characteristics of patients stratified by NCR quartiles are presented in Table 5.

**Table 4:** Association between NCR quartiles and postoperative recurrence of anal fistula.

Characteristic	Model1			Model2			Model3		
	OR	95%CI	Pvalue	OR	95%CI	Pvalue	OR	95%CI	Pvalue
Continuous									
Q1									
Q2	0.334	0.234–0.472	<0.001	0.332	0.232–0.469	<0.001	0.331	0.232–0.468	<0.001
Q3	0.239	0.161–0.348	<0.001	0.237	0.160–0.345	<0.001	0.234	0.158–0.342	<0.001
Q4	0.341	0.239–0.481	<0.001	0.34	0.238–0.479	<0.001	0.339	0.238–0.479	<0.001
*P* for trend	<0.001			<0.001			<0.001		

Model 1: No covariates were adjusted for.

Model 2: Adjusted for age and sex.

Model 3: Adjusted for age, sex, alcohol consumption, smoking status, and body mass index (BMI).

**Table 5:** Baseline characteristics of patients stratified by NCR quartiles.

Characteristic	Level	Q1	Q2	Q3	Q4	p
n		443	443	443	443	
Sex (%)	Female	74 (16.7)	81 (18.3)	64 (14.4)	62 (14.0)	0.261
	Male	369 (83.3)	362 (81.7)	379 (85.6)	381 (86.0)	
Historyofdiabetesmellitus (%)	No	413 (93.2)	419 (94.6)	416 (93.9)	413 (93.2)	0.813
	Yes	30 (6.8)	24 (5.4)	27 (6.1)	30 (6.8)	
Historyofhypertension (%)	No	368 (83.1)	344 (77.7)	361 (81.5)	361 (81.5)	0.206
	Yes	75 (16.9)	99 (22.3)	82 (18.5)	82 (18.5)	
Highanalfistula (%)	No	389 (87.8)	419 (94.6)	419 (94.6)	414 (93.5)	<0.001
	Yes	54 (12.2)	24 (5.4)	24 (5.4)	29 (6.5)	
Multiplefistulatracts (%)	No	357 (80.6)	405 (91.4)	391 (88.3)	396 (89.4)	<0.001
	Yes	86 (19.4)	38 (8.6)	52 (11.7)	47 (10.6)	
Alcohol (%)	No	400 (90.3)	411 (92.8)	399 (90.1)	392 (88.5)	0.186
	Yes	43 (9.7)	32 (7.2)	44 (9.9)	51 (11.5)	
Smoking (%)	No	420 (94.8)	423 (95.5)	410 (92.6)	415 (93.7)	0.261
	Yes	23 (5.2)	20 (4.5)	33 (7.4)	28 (6.3)	
Age [mean (SD)]		38.67 (11.63)	39.37 (12.73)	39.44 (12.33)	38.90 (12.30)	0.747
Height [mean (SD)]		172.40 (7.43)	171.88 (7.52)	171.87 (6.97)	172.63 (7.23)	0.302
Weight [mean (SD)]		75.50 (15.40)	75.14 (14.97)	77.59 (14.40)	77.97 (13.31)	0.005
BMI [mean (SD)]		25.30 (4.41)	25.29 (3.91)	26.18 (4.12)	26.07 (3.59)	<0.001
Neut_pre [mean (SD)]		61.91 (10.98)	61.15 (9.20)	61.91 (9.72)	63.49 (9.26)	0.004
Neut [mean (SD)]		4.18 (2.30)	4.16 (1.82)	4.37 (2.06)	4.57 (1.86)	0.008
Mono_pre [mean (SD)]		6.47 (1.80)	6.07 (1.56)	5.97 (1.44)	5.72 (1.48)	<0.001
Mono [mean (SD)]		0.42 (0.20)	0.40 (0.17)	0.41 (0.19)	0.40 (0.15)	0.343
Baso [mean (SD)]		0.02 (0.01)	0.03 (0.01)	0.03 (0.01)	0.03 (0.02)	0.003
Eos [mean (SD)]		0.14 (0.12)	0.15 (0.13)	0.15 (0.12)	0.15 (0.13)	0.314
MCV [mean (SD)]		89.31 (5.74)	89.62 (4.67)	89.77 (4.94)	89.66 (3.95)	0.528
MPV [mean (SD)]		10.14 (1.27)	10.34 (1.32)	10.36 (1.32)	10.36 (1.23)	0.026
Lymph [mean (SD)]		1.75 (0.62)	1.93 (0.62)	1.95 (0.68)	1.93 (0.63)	<0.001
WBC [mean (SD)]		7.45 (10.18)	7.12 (5.27)	6.88 (3.43)	7.15 (4.49)	0.613
RBC [mean (SD)]		4.76 (0.58)	4.86 (0.58)	4.86 (0.88)	4.90 (0.49)	0.014
PLT [mean (SD)]		236.55 (70.30)	230.76 (59.75)	226.51 (57.82)	231.74 (48.50)	0.096
Hb [mean (SD)]		141.56 (18.63)	145.37 (14.59)	147.07 (15.49)	148.99 (13.75)	<0.001
hs_CRP [mean (SD)]		17.30 (20.66)	6.02 (2.83)	3.54 (1.90)	1.78 (0.82)	<0.001
NLR [mean (SD)]		2.80 (2.31)	2.43 (1.74)	2.60 (2.29)	2.73 (2.36)	0.062
PLR [mean (SD)]		154.09 (81.96)	131.40 (57.41)	129.86 (62.84)	132.24 (52.57)	<0.001
SII [mean (SD)]		674.80 (648.67)	569.98 (469.29)	602.16 (664.41)	635.32 (547.95)	0.052
SIRI [mean (SD)]		1.38 (1.94)	1.06 (1.10)	1.22 (2.32)	1.14 (1.24)	0.041
AISI [mean (SD)]		343.58 (515.54)	255.47 (327.28)	293.72 (639.90)	268.73 (311.17)	0.028
LCR [mean (SD)]		0.17 (0.12)	0.37 (0.16)	0.65 (0.32)	1.24 (0.56)	<0.001

## 4 Discussion

This study developed and interpreted a GBM-based model for predicting postoperative recurrence of anal fistula using SHAP analysis. WBC, RBC, hs-CRP, and NCR showed consistent predictive value, and RCS analysis revealed a nonlinear association between NCR and recurrence risk.

Postoperative recurrence of anal fistula remains a major challenge in colorectal surgery. Recurrence has been associated with fistula complexity, recurrent or missed tracts, previous anorectal abscess, diabetes, and surgical approach (4, 12, 15). While these factors are mainly anatomical or operative, systemic inflammation has been less explored in recurrence prediction. Our findings suggest that routine blood cell parameters and inflammation-related indices may help improve postoperative risk stratification.

In this study, WBC and hs-CRP were independently associated with postoperative recurrence. This finding is clinically plausible because both indicators reflect inflammatory activity and infection burden. Persistent preoperative inflammation may indicate active local infection, delayed wound healing, or incomplete control of the underlying inflammatory process. Compared with anatomical variables alone, inflammatory markers may better capture the systemic host response, which is difficult to assess from operative findings.

A strength of this study was the inclusion of several composite inflammation-related indices, including NCR, LCR, SII, SIRI, and AISI. SII, SIRI, and AISI have been reported as systemic inflammatory and immune-related markers (16–18) and LCR and NCR have been evaluated in surgical and prognostic settings (19–21). However, their use for predicting postoperative recurrence of anal fistula remains limited. Among these indices, NCR showed the most consistent predictive value. It was identified as an independent predictor by multivariable logistic regression, retained by LASSO regression, ranked among the important variables in SHAP analysis, and showed a significant nonlinear association with recurrence risk in RCS analysis. These findings suggest that NCR may be a practical marker for recurrence risk stratification.

The association between NCR and recurrence warrants attention. NCR integrates neutrophil count and CRP and may reflect the balance between cellular inflammatory response and systemic inflammatory burden. A lower NCR may indicate relatively higher CRP levels and a stronger inflammatory state, which may impair postoperative tissue repair. RCS analysis showed that recurrence risk decreased markedly as NCR increased from low levels, but this reduction plateaued at higher values. Thus, NCR should not be interpreted as a purely linear protective marker. Instead, it may be more useful as a nonlinear risk stratification indicator, particularly for identifying patients with low NCR who may require closer follow-up.

RBC was also an independent predictor and contributed to the final GBM model, although its clinical relevance to anal fistula recurrence is less direct than that of WBC and hs-CRP. RBC may reflect hematologic status, tissue oxygenation, or perioperative physiological reserve, but this finding should be interpreted cautiously because of possible unmeasured confounding or population-specific characteristics. Further studies are needed to clarify whether RBC has direct biological relevance or mainly serves as a model-based predictive variable.

Compared with traditional regression models, GBM can better capture nonlinear effects and interactions among predictors. In this study, GBM showed stable discrimination, balanced classification performance, acceptable calibration, and potential clinical net benefit, supporting its use as an auxiliary tool for individualized recurrence risk assessment. SHAP analysis improved model interpretability by clarifying each variable's contribution. Although age had the highest SHAP importance, it was not an independent risk factor and should therefore be interpreted as model-based predictive information rather than evidence of causality. In contrast, WBC, RBC, hs-CRP, and NCR were supported by both regression and machine learning analyses, suggesting stable predictive value.

Published clinical prediction tools for postoperative outcomes of anal fistula remain limited, and direct comparison across studies is difficult because of substantial differences in patient populations, operative procedures, predictor availability, outcome definitions, and assessment time points. For example, an MRI-based nomogram developed in 200 patients with complex cryptoglandular anal fistulas predicted early postoperative fistula healing, with AUCs of 0.880 and 0.847 in the training and testing cohorts, respectively (22). However, that model incorporated quantitative preoperative and postoperative MRI parameters and focused on early healing rather than long-term recurrence. Similarly, the Garg scoring system combines clinical assessment with postoperative MRI findings to predict long-term healing; in a prospective validation study of 50 patients, it showed a high positive predictive value for healing but lower accuracy for identifying nonhealing (23). These tools therefore address different clinical questions and require imaging information that may not be routinely or uniformly available.

Against this limited and heterogeneous background, the AUC of 0.712 observed in our ward-based internal validation cohort indicates moderate rather than high discrimination. The potential advantage of the present model is its reliance on routinely available clinical and laboratory variables, which may facilitate preoperative or early postoperative risk estimation without requiring specialized imaging-derived measurements. Nevertheless, an AUC of approximately 0.71 alone is not sufficient to support major treatment decisions. The model should therefore be regarded as an adjunctive risk-stratification tool rather than a replacement for clinical assessment, fistula anatomy evaluation, imaging, or surgeon judgment. In patients with a relatively high predicted risk, the model may support closer postoperative surveillance, more frequent wound assessment, and selective imaging when clinically indicated. It should not be used alone to determine operative technique, reoperation, or other invasive interventions. Although decision curve analysis suggested potential net benefit within low-to-moderate threshold probability ranges, prospective impact studies are required to determine whether model-guided follow-up improves outcomes compared with usual care.

To facilitate model implementation, we deployed the final GBM model as an interactive web-based calculator using the Shiny framework. The application enables standardized entry of the 11 model predictors and automatically returns an individualized probability of postoperative recurrence. Because most predictors are routinely available in the electronic medical record, a future clinical decision-support module could automatically retrieve these variables from the hospital information system and generate recurrence-risk estimates without manual data entry. However, before electronic medical record integration or routine deployment, predictor definitions and measurement units should be standardized, rules for missing or implausible values should be prespecified, and the algorithm and classification threshold should be locked. External calibration, prospective workflow evaluation, and monitoring for model-performance drift would also be required. Therefore, the current web application should be regarded as an adjunctive research tool rather than a fully validated clinical decision-support system.

Several limitations should be acknowledged. First, patients from wards 15 and 16 were reserved as a ward-based internal validation cohort and were managed by clinical teams separate from those responsible for the development wards. This unit-based separation provided a more stringent assessment of between-team reproducibility than a purely random patient-level split. Nevertheless, all patients originated from the same institution, were treated during the same study period, and shared the same laboratory systems and institutional management protocols. Therefore, this cohort represents ward-based internal validation rather than true external validation. The present findings do not establish transportability to other hospitals, regions, or healthcare systems, and independent multicenter and prospective temporal validation remains necessary. Second, all patients included in this study underwent fistulectomy; therefore, operative technique showed no variation within the study population and was not included as a candidate predictor. Although the uniform operative approach reduced heterogeneity associated with differences in surgical procedures, it limits the applicability of the model to patients treated with other techniques. Accordingly, the model should not be directly extrapolated to patients undergoing fistulotomy, seton-based procedures, LIFT, or advancement flap without further validation. In addition, several clinically important variables, including Parks classification, detailed MRI findings, postoperative wound-management factors, and surgeon-level experience, were unavailable or incompletely recorded. Their omission may have introduced residual confounding and limited the predictive performance and transportability of the model. MRI is an important source of anatomical and quantitative imaging information in anal fistula because it can identify internal openings, secondary tracts, horseshoe extensions, supralevator involvement, occult abscesses, and relationships with the sphincter complex that may influence postoperative outcomes (24, 25). However, preoperative MRI was not routinely performed in all patients in this retrospective cohort, and the proportion of missing MRI data was substantial. MRI-derived variables were therefore not included in the present model to avoid excessive sample loss and potential selection bias. Future prospective studies will standardize MRI acquisition, expand the cohort of patients with complete imaging data, and integrate conventional MRI features and radiomic signatures with clinical and laboratory variables to develop a multimodal recurrence-prediction model. Third, because recurrence occurred in a relatively small proportion of patients, the model showed limited positive predictive performance. Therefore, its current use may be more appropriate for risk stratification and follow-up planning. Finally, although SHAP analysis improved interpretability, causality could not be established, and the observed associations require prospective validation.

## 5 Conclusion

WBC, RBC, hs-CRP, and NCR were independently associated with postoperative recurrence of anal fistula. The GBM model based on LASSO-selected variables showed stable predictive performance and acceptable clinical utility. The incorporation of routine blood cell parameters and inflammation-related composite indices, particularly NCR, provided additional value beyond conventional clinical factors. SHAP and RCS analyses further improved model interpretation and revealed a nonlinear association between NCR and recurrence risk. These findings may help identify patients at increased risk of recurrence and support individualized postoperative follow-up. Because the model was developed exclusively in patients undergoing fistulectomy, its applicability to patients treated with other operative procedures remains uncertain. Independent multicenter validation is required before broader clinical implementation.

## Data Availability

The raw data supporting the conclusions of this article will be made available by the authors, without undue reservation.

## References

[1] Reza L Gottgens K Kleijnen J Breukink S Ambe PC Aigner F. European Society of coloproctology: guidelines for diagnosis and treatment of cryptoglandular anal fistula. Colorectal Dis. (2024) 26(1):145–96. 10.1111/codi.16741 38050857

[2] Gandhi ND Alibo EO Gipe JH. Anal abscess and Fistula. Surg Clin North Am. (2026) 106(1):51–63. 10.1016/j.suc.2025.08.012 41241453

[3] Dong S Chen B Zhang J. Study on the factors influencing the prognosis after perianal abscess surgery. BMC Gastroenterol. (2023) 23(1):334. 10.1186/s12876-023-02959-1 37759161 PMC10537581

[4] Khan S Kotcher R Herman P Wang L Tessler R Cunningham K. Predictors of recurrence and long-term patient reported outcomes following surgical repair of anal fistula, a retrospective analysis. Int J Colorectal Dis. (2024) 39(1):37. 10.1007/s00384-024-04602-1 38466439 PMC10927793

[5] Andreou C Zeindler J Oertli D Misteli H. Longterm outcome of anal fistula—a retrospective study. Sci Rep. (2020) 10(1):6483. 10.1038/s41598-020-63541-3 32300218 PMC7162908

[6] Li S Xu H Wang W Gao H Li H Zhang S. The systemic inflammation response index predicts survival and recurrence in patients with resectable pancreatic ductal adenocarcinoma. Cancer Manag Res. (2019) 11:3327–37. 10.2147/CMAR.S197911 31114368 PMC6489619

[7] Jiang Y Luo B Lu W Chen Y Peng Y Chen L. Association between the Aggregate Index of systemic inflammation and clinical outcomes in patients with acute myocardial infarction: a retrospective study. J Inflamm Res. (2024) 17:7057–67. 10.2147/JIR.S481515 39377046 PMC11457786

[8] Ullah W Basyal B Tariq S Almas T Saeed R Roomi S. Lymphocyte-to-C-Reactive protein ratio: a novel predictor of adverse outcomes in COVID-19. J Clin Med Res. (2020) 12(7):415–22. 10.14740/jocmr4227 32655735 PMC7331862

[9] Martinez-Mier G Carbajal-Hernández R López-García M Uría-Torija T Reyes-Ruiz JM Solórzano-Rubio JR. Prospective analysis of preoperative C-reactive protein and neutrophil-to-lymphocyte ratio as predictors of postoperative complications in bile duct injury repair. Updates Surg. (2025) 77(1):47–56. 10.1007/s13304-024-02054-4 39692980

[10] Yuan X Xu Q Du F Gao X Guo J Zhang J. Development and validation of a model to predict cognitive impairment in traumatic brain injury patients: a prospective observational study. eClinicalMed. (2025) 80:103023. 10.1016/j.eclinm.2024.103023 PMC11753911 39850016

[11] Ramírez Pedraza N Pérez Segovia AH Garay Mora JA Techawatanaset K Bowman AW Cruz Marmolejo MA. Perianal Fistula and abscess: an imaging guide for beginners. Radiographics. (2022) 42(7):E208–9. 10.1148/rg.210142 36083806

[12] Gaertner WB Burgess PL Davids JS Lightner AL Shogan BD Sun MY. The American society of colon and rectal surgeons clinical practice guidelines for the management of anorectal abscess, Fistula-in-ano, and rectovaginal Fistula. Dis Colon Rectum. (2022) 65(8):964–85. 10.1097/DCR.0000000000002473 35732009

[13] Wang D Zhang C Li Z Zheng Z Chen A Fang Y. Postoperative recurrence prediction model for perianal abscess using machine learning algorithms. Front Public Health. (2025) 13:1722109. 10.3389/fpubh.2025.1722109 41393021 PMC12700084

[14] Celik O Gundogan E Turk G Carkit S Ertan T. Can inflammatory Index parameters be an indicator of complexity in perianal Fistula? J Surg Res. (2025) 314:544–50. 10.1016/j.jss.2025.07.059 40857865

[15] Emile SH. Recurrent anal fistulas: when, why, and how to manage? World J Clin Cases. (2020) 8(9):1586–91. 10.12998/wjcc.v8.i9.1586 32432136 PMC7211523

[16] Hu B Yang X-R Xu Y Sun Y-F Sun C Guo W. Systemic immune-inflammation index predicts prognosis of patients after curative resection for hepatocellular carcinoma. Clin Cancer Res. (2014) 20(23):6212–22. 10.1158/1078-0432.CCR-14-0442 25271081

[17] Qi Q Zhuang L Shen Y Geng Y Yu S Chen H. A novel systemic inflammation response index (SIRI) for predicting the survival of patients with pancreatic cancer after chemotherapy. Cancer. (2016) 122(14):2158–67. 10.1002/cncr.30057 27152949

[18] Zinellu A Collu C Nasser M Paliogiannis P Mellino S Zinellu E. The Aggregate Index of systemic inflammation (AISI): a novel prognostic biomarker in idiopathic pulmonary fibrosis. J Clin Med. (2021) 10(18):4134. 10.3390/jcm10184134 34575245 PMC8466198

[19] Utsumi M Inagaki M Kitada K Tokunaga N Kondo M Sakurai Y. Preoperative lymphocyte-to-C-reactive protein ratio predicts hepatocellular carcinoma recurrence after surgery. Ann Surg Treat Res. (2022) 103(2):72–80. 10.4174/astr.2022.103.2.72 36017137 PMC9365642

[20] Shaukat W Baig AM Ali Z Kumari K Tariq T Soomro AK. Prognostic value of C-reactive protein and neutrophil-to-lymphocyte ratio in predicting postoperative infections after gastrointestinal surgery: a meta-analysis. Cureus. (2025) 17(8):e91123. 10.7759/cureus.91123 41018345 PMC12468170

[21] Eyvaz K Dinçer O Aydemir E Kazim-Kazan M AkguÓl N Aslaner A. Preoperative neutrophil-to-C-reactive protein ratio as a predictor of post-operative complications of pancreas cancer. Cir Cir. (2024) 92(4):481–6. 10.24875/CIRU.22000544 39079242

[22] Xu H Xiao G-Z Zheng Y-H Fu Y-J Zhong S-L Ren D-L. A magnetic resonance imaging-based decision-making tool for predicting complex anal fistulas healing in the early postoperative period. BMC Gastroenterol. (2023) 23(1):372. 10.1186/s12876-023-02963-5 37907854 PMC10617037

[23] Dawka S Yagnik VD Kaur B Menon GR Garg P. Garg scoring system to predict long-term healing in cryptoglandular anal fistulas: a prospective validation study. Ann Coloproctol. (2024) 40(5):490–7. 10.3393/ac.2022.00346.0049 36217811 PMC11532377

[24] Teymouri A Keshvari A Ashjaei A Ahmadi Tafti SM Salahshour F Khorasanizadeh F. Predictors of outcome in cryptoglandular anal fistula according to magnetic resonance imaging: a systematic review. Health Sci Rep. (2023) 6(6):e1354. 10.1002/hsr2.1354 37359408 PMC10286857

[25] Konan A Mr O Mn Ö. The contribution of preoperative MRI to the surgical management of anal fistulas. Diagn Interv Radiol. (2018) 24(6):321–7. 10.5152/dir.2018.18340 30272562 PMC6223824

