# Supplementary material for: Development and validation of a SHAP-interpretable GBM model for predicting postoperative recurrence of anal Fistula
Source: Front Surg. 2026 Aug 26;13:1911496. doi: 10.3389/fsurg.2026.1911496 (PMC13554486; doi:10.3389/fsurg.2026.1911496)
Supplement: Supplementary file 1 [file Datasheet1.pdf]

Figure S1. Confusion matrices of the GBM model.

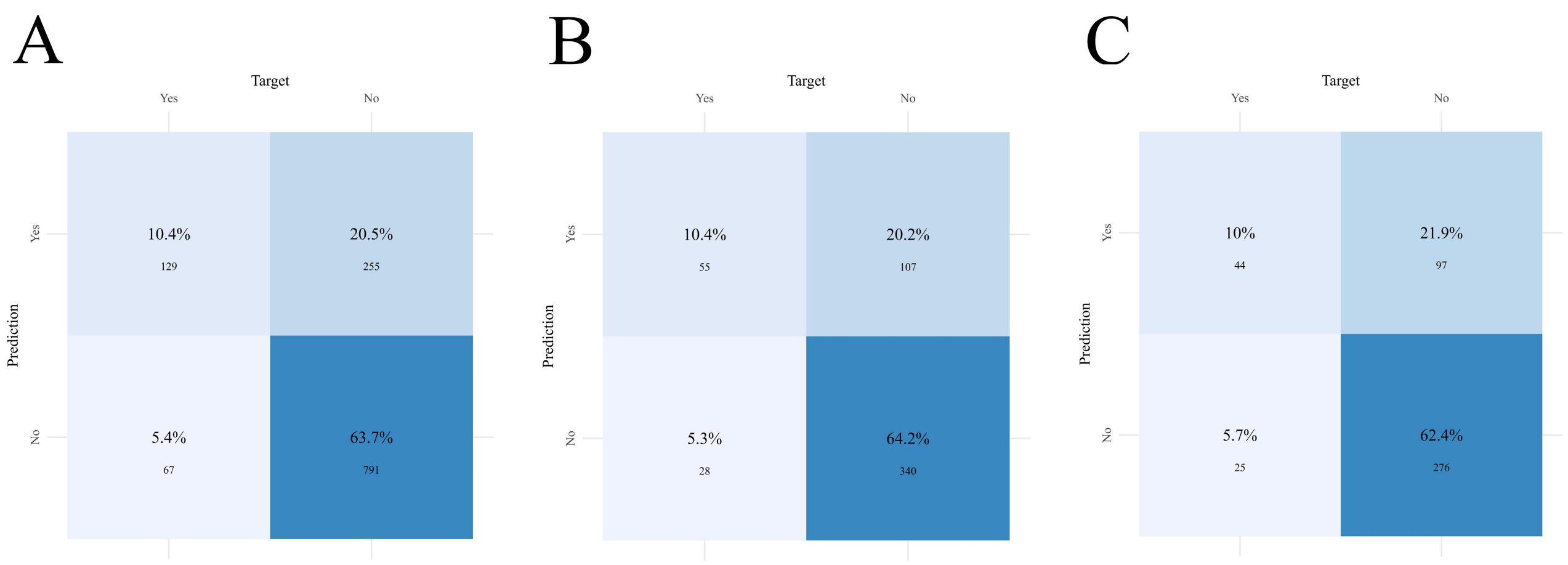

Figure S1. Confusion matrices of the final GBM model in the training, testing, and validation cohorts. GBM, gradient boosting machine.
