# Supplementary material for: Development and validation of a SHAP-interpretable GBM model for predicting postoperative recurrence of anal Fistula
Source: Front Surg. 2026 Aug 26;13:1911496. doi: 10.3389/fsurg.2026.1911496 (PMC13554486; doi:10.3389/fsurg.2026.1911496)
Supplement: Supplementary file 2 [file Datasheet2.pdf]

Figure S2. Additional SHAP interpretation plots.

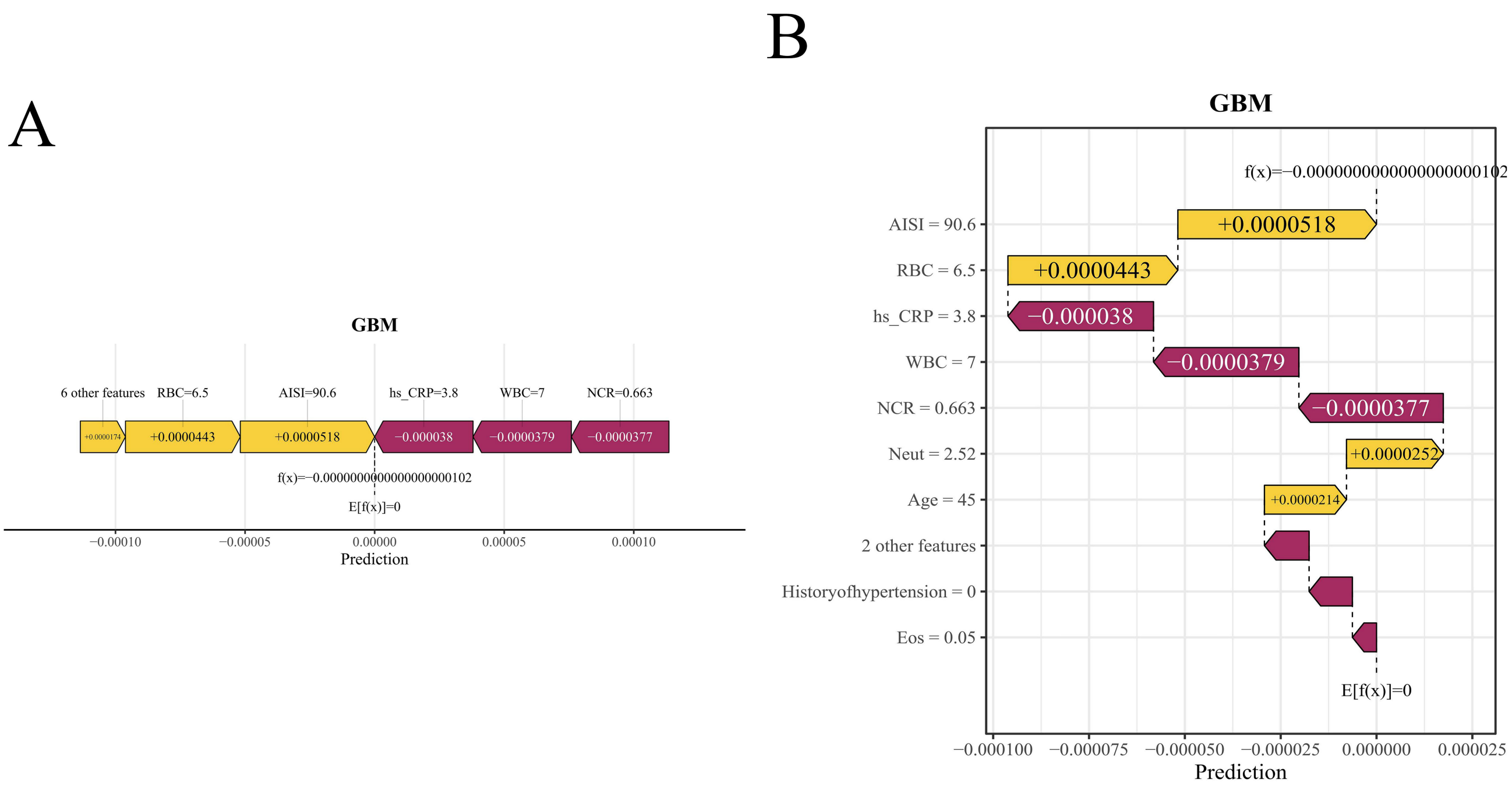

Figure S2. Additional SHAP interpretation plots of the final GBM model, including force plot, waterfall plot, and partial dependence plot. SHAP, Shapley additive explanations; GBM, gradient boosting machine.
